# Evaluation of the 3D Printing Accuracy of a Dental Model According to Its Internal Structure and Cross-Arch Plate Design: An In Vitro Study

**DOI:** 10.3390/ma13235433

**Published:** 2020-11-28

**Authors:** Seung-Ho Shin, Jung-Hwa Lim, You-Jung Kang, Jee-Hwan Kim, June-Sung Shim, Jong-Eun Kim

**Affiliations:** Department of Prosthodontics, Yonsei University College of Dentistry, Yonsei-ro 50-1, Seodaemun-gu, Seoul 03722, Korea; shin506@prostholabs.com (S.-H.S.); erin0313@prostholabs.com (J.-H.L.); kyj@yuhs.ac (Y.-J.K.); jee917@yuhs.ac (J.-H.K.); jfshim@yuhs.ac (J.-S.S.)

**Keywords:** 3D printing, CAD/CAM, additive manufacturing, internal structure, trueness, precision

## Abstract

The amount of photopolymer material consumed during the three-dimensional (3D) printing of a dental model varies with the volume and internal structure of the modeling data. This study analyzed how the internal structure and the presence of a cross-arch plate influence the accuracy of a 3D printed dental model. The model was designed with a U-shaped arch and the palate removed (Group U) or a cross-arch plate attached to the palate area (Group P), and the internal structure was divided into five types. The trueness and precision were analyzed for accuracy comparisons of the 3D printed models. Two-way ANOVA of the trueness revealed that the accuracy was 135.2 ± 26.3 µm (mean ± SD) in Group U and 85.6 ± 13.1 µm in Group P. Regarding the internal structure, the accuracy was 143.1 ± 46.8 µm in the 1.5 mm-thick shell group, which improved to 111.1 ± 31.9 µm and 106.7 ± 26.3 µm in the roughly filled and fully filled models, respectively. The precision was 70.3 ± 19.1 µm in Group U and 65.0 ± 8.8 µm in Group P. The results of this study suggest that a cross-arch plate is necessary for the accurate production of a model using 3D printing regardless of its internal structure. In Group U, the error during the printing process was higher for the hollowed models.

## 1. Introduction

Dental models have been produced to reproduce patient dental information outside the oral cavity for treatment processes such as consultation, diagnosis, and prosthesis fabrication [1]. However, the traditional plaster model produced after making intraoral impressions using alginate or silicone impression material can be damaged, either during the transportation or storage of the impression body, and the plaster model itself [1]. Moreover, the need to store various models made during long-term orthodontic treatment or prosthetic treatment can result in difficulties associated with physical space limitations [2]. Recent improvements in dental computer-aided design/computer-aided manufacturing (CAD/CAM) systems have made it possible to digitize information about the oral cavity using an intraoral scanner or scanning a model using model scanner [3]. The obtained digitized data can be stored, retrieved, transmitted, and analyzed using various types of digital software [4,5]. In the diagnosis process for orthognathic surgery or implant surgery, cone beam computed tomography (CBCT) data and digital model data can be aligned, and this can be used to establish a surgery or treatment plan on digital software as well [6,7]. However, despite these benefits of digital models, physical models are still required in many clinical situations, and so digital data must be manufactured accurately when a physical dental model is needed [3,8]. 

The methods for producing products from digital data can be broadly divided into additive manufacturing (AM) and subtractive manufacturing (SM) [9]. SM has the following disadvantages: directly milling materials with the milling bur [10] means that precise areas smaller than the diameter of the milling bur cannot be processed [11], and it is wasteful when a large amount of material needs to be discarded [12]. These aspects make SM an unfavorable method for making dental models. AM methods such as digital light processing (DLP) and stereolithography (SLA) have recently been widely used [13], and 3D printing has become widely used in the dental field due to improvements in the accuracy and speed of printing [8]. A 3D printer using AM converts the designed CAD file into slice data, organizes it by layer, and creates the desired shape [14]. AM uses the smallest possible amount of material and can be used to manufacture objects with complex internal shapes [2,15].

When creating a dental model using 3D printing, it is very important that the scanned data are accurately manufactured and reproduced [4]. When using a printed model to evaluate occlusion or fabricate a prosthesis, errors in the model can affect the accuracy of the produced prosthesis [16]. Previous studies of the accuracy of 3D printed dental models have included comparisons and evaluations of the accuracies of conventional gypsum models and models produced using SM [17,18,19], and comparisons of the accuracy of the produced model have been made according to the 3D printer type [20]. Other studies have investigated parameters that mainly affect the accuracy of printed components, such as the type of resin used, printing angle, layer thickness, and temperature [8,21,22,23,24,25,26]. It has been reported that DLP and SLA printers are the most suitable for dental 3D printing, since they are more precise than printers based on fused deposition modeling [16,27].

The large amount of photocured resin required to manufacture a dental model is expensive, and so various efforts have been made to reduce the resin material consumed [3]. If a part such as the palate or tongue is unnecessary to the purpose of a diagnosis or treatment, it can be removed during the premodeling process and then not printed, producing a hollow design inside the model [28,29]. Such a modeling strategy can also reduce the printing time required for the 3D printing process [2,30]. A previous study that controlled the internal structure compared hollow and nonhollow models [31]. However, no previous studies have applied various hollowing methods to determine changes according to the amount of hollowing or forming a lattice structure internally, or investigated internal structure changes and interactions with a cross-arch plate.

Therefore, the present study was designed to evaluate the accuracy of 3D printed models according to the presence or absence of a cross-arch plate, the internal filling rate, and the thickness of the shell. The following null hypotheses were established: (1) the accuracy of a 3D printed model does not vary with the presence and absence of a cross-arch plate, and (2) the accuracy does not vary with the shell thickness or interior structure design of a 3D printed model.

## 2. Materials and Methods 

**Figure 1:**
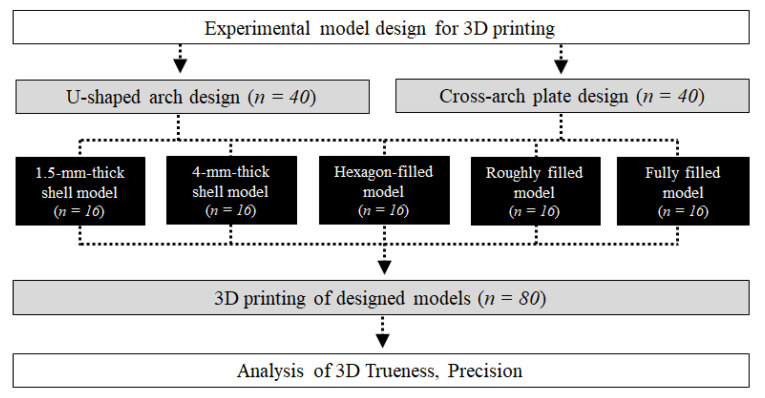
Flowchart of the overall experimental process of this study.

**Figure 2:**
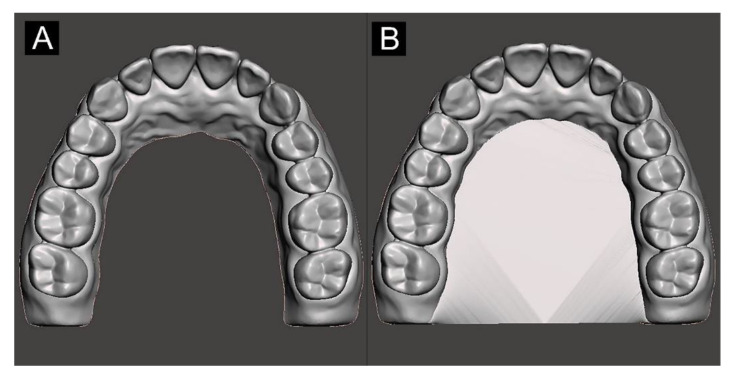
Experimental model designs: (**A**) U-shaped arch and (**B**) cross-arch plate.

The model design and overall experimental workflow of this study are illustrated in Figure 1. To design the model in each group, a maxillary tooth dentiform model (D85DP-500B.1, Nissin, Kyoto, Japan) was scanned using a tabletop scanner (Identica T500, Medit, Seoul, Korea), with the obtained data exported in the Standard Triangulated Language (STL) digital file format. The converted design file was then imported into the modeling software (Meshmixer, Autodesk, San Rafael, CA, USA), the palate and other unnecessary structures were deleted, and the U-shaped model (Group U) was designed. The cross-arch plate model (Group P) was designed by attaching a 1.5 mm-thick cross-arch plate at the position of the palate in Group U (Figure 2).

**Figure 3:**
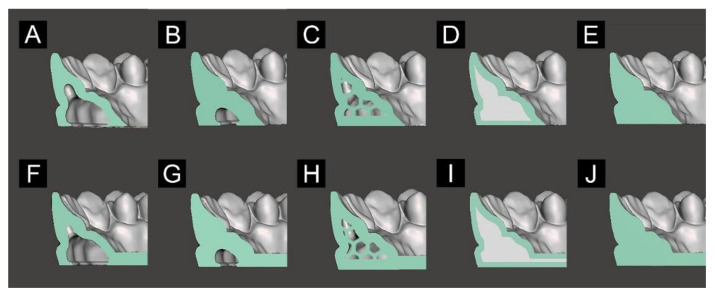
Designs of the 3D printed models: (**A**,**F**) hollow model with a 1.5 mm-thick shell, (**B**,**G**) hollow model with a 4 mm-thick shell, (**C**,**H**) hexagon-filled model, (**D**,**I**) roughly filled model, (**E**,**J**) fully filled model. (**A**–**E**) U-shaped model design; and (**F**–**J**) cross-arch plate model design.

Based on the designed files for Groups U and P, 3D modeling software (Meshmixer, Autodesk, San Rafael, CA, USA; Rhino 5, Robert McNeel & Associates, Seattle, WA, USA) was used to model the internal structure of the model area excluding the plate area in various ways (Figure 3). For the 1.5 mm hollow model (Figure 3A), the hollowing function was applied after designating the shell thickness of the model as 1.5 mm (Figure 3A,F). The 4 mm hollow model was the same as the 1.5 mm hollow model except that it had a 4 mm thick shell (Figure 3B,G). For the hexagon-filled model, after designing a hollow model with a shell thickness of 1.5 mm, the hexagon structure was processed and arranged according to the shape of the hollow inner surface of the model (Figure 3C,H). The hexagon structure was produced by designing a hexagon shape with a side length of 2 mm using Adobe Illustrator CS (Adobe, San Jose, CA, USA), and then using the extrude closed planar curve function of CAD software (Rhino 5, Robert McNeel & Associates, Seattle, WA, USA). The roughly filled model used a solid model that completely filled the interior (i.e., no empty space), and a selective pixel application technique was applied during 3D printing by using the pixel-dimming function of slicing open-source software (NanoDLP) to adjust the illuminance of pixels on the liquid crystal display (LCD) screen when 3D printing was applied (Figure 3D,I). A 50% pixel opening setting was applied in the option setting of the selective pixel application. A fully filled model that was completely filled internally with no empty space was then printed in 3D (Figure 3E,J).

Eight models for each group were printed using the photopolymer resin (MAZIC^®^D, Vericom, Chuncheon, Korea) and a DLP 3D printer (Phrozen Shuffle, Phrozen, Hsinchu, Taiwan). The thickness of each printing layer was set to 100 μm, and a printing support structure was attached to the bottom of the model. Table 1 lists the amounts of resin consumed for the different printed models.

All of the printed models were washed with 90% isopropyl alcohol in a washing machine (Formwasher, Formlabs, Somerville, MA, USA) and then polymerized using an ultraviolet post-curing unit (CureM D102, Sona Global, Seoul, Korea) according to the manufacturer’s instructions. 

Each printed model was scanned with a tabletop 3D scanner, and the scanned data were exported as an STL file and sent for 3D inspection software (Geomagic Control X, 3D Systems, Rock Hill, SC, USA). Removing the lower part of the scan data up to the height of the cross-arch plate design, only the tooth area and the surrounding gingival area were designated equally. Trueness was calculated by overlapping all the data of each group with the model design reference data, and precision was calculated by overlapping the data in each group in a pairwise manner. Surface deviation data were calculated using root-mean-square estimation (RMSE) to perform overall volume comparisons [32]. The RMSE was calculated using the following Equation (1):

(1) $$\mathrm{RMSE}\;=\frac{1}{\sqrt{n}}\cdot \sqrt{\overset{n}{\underset{i=1}{\sum }}{\left(x_{1,i}-x_{2,i}\right)}^{2}}$$

All scan data were analyzed statistically to compare the trueness and precision values between groups, and the mean discrepancy obtained from the comparisons of two data sets was used for statistical analysis. A 3D comparative analysis was used to determine the accuracy of the model output; that is, the accuracy of the scanner comprised a combination of trueness (measure of the differences from the reference model) and precision (measure of the similarity between measurements).

Statistical analyses of the results in each group were performed using standard statistical software (SPSS version 25.0, SPSS, IBM SPSS Statistics, Armonk, USA). All of the acquired data were subjected to Levene’s test to evaluate homoscedasticity, and the Shapiro–Wilk normality test was used to test for the presence of normality. Differences between the groups according to the presence or absence of a cross-arch plate and the internal structure were analyzed using the two-way ANOVA test, with the one-way ANOVA test used to verify the difference in accuracy according to the internal structure in each of Groups P and U. The Bonferroni test was used as a posttest. The student’s *t*-test was used to compare differences between with and without a cross-arch plate in groups with the same internal structure. The significance cutoff in all tests was α = 0.05.

## 3. Results

**Figure 4:**
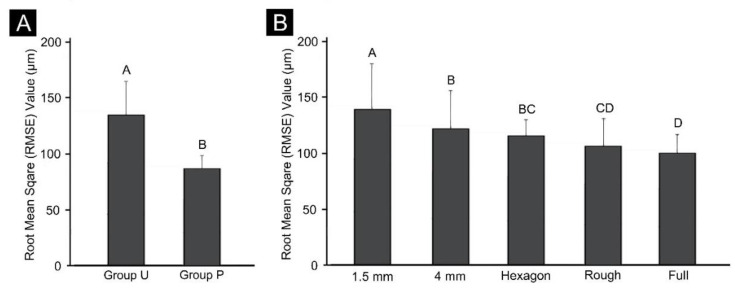
Results of the two-way ANOVAs of trueness (**A**) between Groups U and P, and (**B**) between the groups with different internal structures. Uppercase letters indicate significant differences. Data are the mean and SD values.

Figure 4 shows the results of two-way ANOVAs of trueness according to the presence or absence of a cross-arch plate and differences in the internal structure. The deviation was 91.0 ± 7.8 μm (mean ± SD) in Group P and 139.1 ± 25.4 μm in Group U, and the accuracy was significantly higher when a cross-arch plate was present (F = 376.5, *p* < 0.001). The internal structure also significantly affected the accuracy (F = 23.4, *p* < 0.001). The accuracy was low in the 1.5 mm and 4 mm-thick shell models with a hollow internal structure, and high in the models in which the internal structure was not hollowed out (e.g., fully filled model). The interaction between the two factors (F = 15.5, *p* < 0.001) was also found to have a significant effect on trueness.

**Figure 5:**
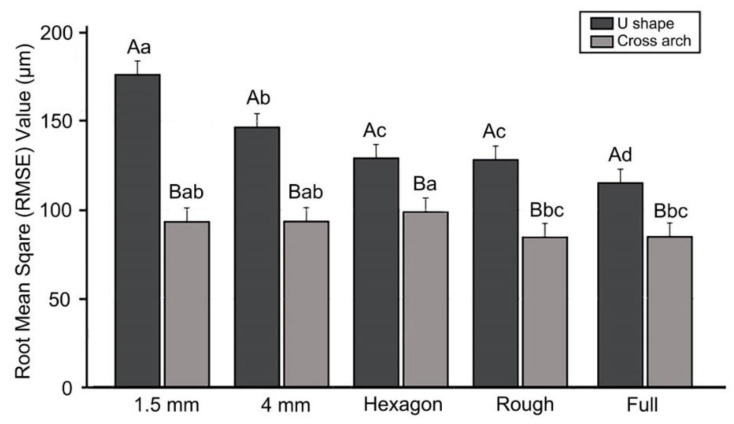
Results of the trueness analysis between Groups P and U. Lowercase letters indicate significant differences between internal structures within the Groups U or P each, and uppercase letters indicate significant differences between Groups P and U within the same internal-structure.

In Group U, the overall RMSE value was high, and it differed markedly depending on the internal structure, being the highest in the 1.5 mm thick model, followed by the 4 mm thick model. In the 1.5 mm thick model, the accuracy was higher in the group filled with the hexagon shape, and highest in the fully filled model. In Group P, the internal structure had lower effects than in Group U, with the accuracy being significantly higher in the roughly filled and fully filled groups (Figure 5). 

**Figure 6:**
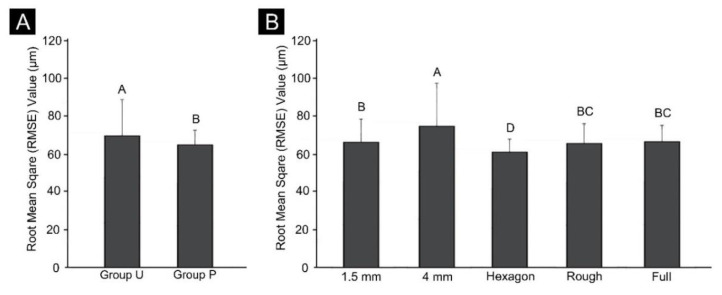
Results of the two-way ANOVA of precision (**A**) between Groups P and U and (**B**) between each internal-structure group. Uppercase letters indicate significant differences. Data are the mean and SD values.

Figure 6 shows the results of the two-way ANOVA of precision according to the presence or absence of a cross-arch plate and differences in internal structure. The deviation was 62.97 ± 9.8 μm in Group P and 71.6 ± 16.81 μm in Group U, and the accuracy was significantly higher in the presence of a cross-arch plate (F = 31.1, *p* < 0.001). The internal structure also significantly affected the accuracy (F = 7.5, *p* < 0.001). The output stability was low for the model with a hollow internal structure (e.g., the 4 mm-thick shell model), and high in the hexagon-filled model. The interaction between the two factors was also found to have a significant effect on precision (F = 3.618, *p* = 0.006).

**Figure 7:**
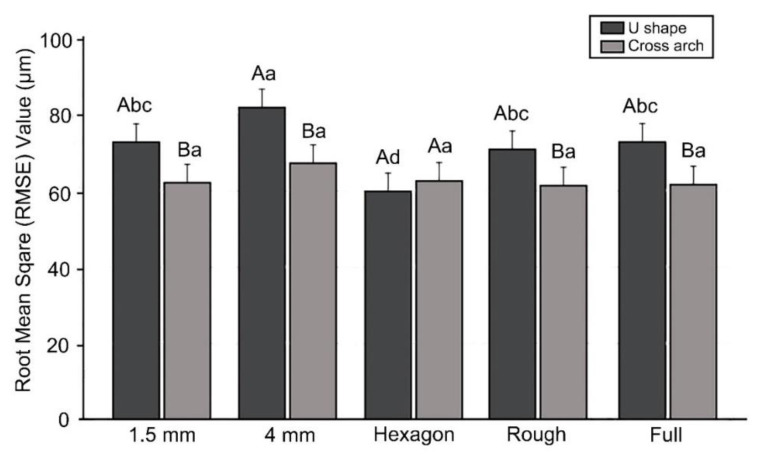
Precision comparison between Groups P and U. Lowercase letters indicate significant differences between internal structures within the Groups U or P each, and uppercase letters indicate significant differences between Groups P and U within the same internal-structure.

The precision did not differ significantly with the internal structure in Group P, while in Group U, there were significant differences in the 1.5 mm hollow model, the roughly filled model, and the fully filled model, and in the 4 mm hollow model and the hexagon-filled model. In Group P, the precision was lowest in the 4 mm hollow model and highest in the roughly filled and fully filled models. In Group U, the precision was the highest in the hexagon-filled model and lowest in the 4 mm hollow model (Figure 7).

**Figure 8:**
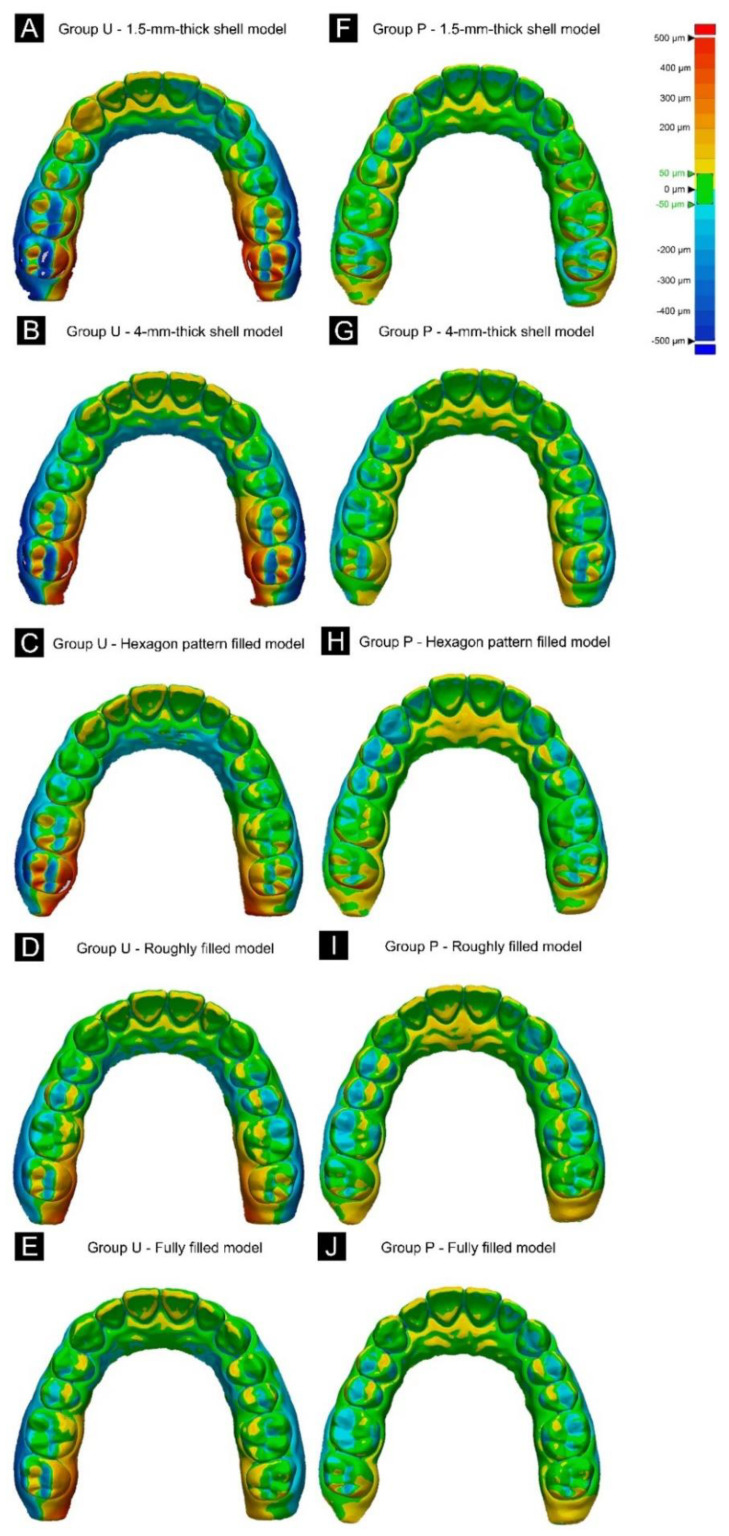
3D color maps of the trueness in (**A**–**E**) Groups U and (**F**–**J**) P. The contraction occurred mainly in the lingual direction in the posterior region in Group U, whereas the overall model was stable in Group P. (**A**,**F**) 1.5mm-thick shell model. (**B**,**G**) 4mm-thick shell model. (**C**,**H**) Hexagon pattern filled model. (**D**,**I**) Roughly filled model. (**E**,**J**) Fully filled model.

3D color maps were used to visualize the characteristic elements of each group (Figure 8). In Group U, the contraction of the arch around the left and right posterior teeth occurred in the lingual direction. In contrast, Group P with a cross-arch plate structure had relatively stable output results. 

## 4. Discussion

This study confirmed that the presence or absence of a cross-arch plate and differences in the internal structure resulted in significant variations in the characteristics of 3D printed models produced using the DLP method. In the trueness analysis of Groups U and P, the overall error was large for Group U, whereas the output result was stable for Group P. The color-map analysis of model overlap revealed that the error in Group U manifested in the model contraction on both lingual sides in the posterior region. Group P, with a cross-arch support plate, was found to be stable. It seems that the shrinkage in the polymerization process is caused by differences in the overall volume in Group U [33,34]. Therefore, the first null hypothesis was rejected. Both 3D printing and post-curing are processes involving the polymerization of a photopolymer resin, and it has been reported that shrinkage during this process affects the dimensional accuracy of produced objects [35,36,37]. In Group U, only the support structure for 3D printing could resist shrinkage, whereas in Group P, the presence of a cross-arch stabilization structure is thought to have resulted in the overall volume stability during each curing process. Thus, the second null hypothesis was also rejected. Camardella et al. [36] also concluded that there are differences in output stability according to various types of base shapes. Among the U-shaped models that those authors investigated, the average interdental distance differed by −0.7 mm when compared with the model group with a general base, and the model group that included a cross bar was arranged in a U shape, and there was no significant difference from the group with the general base. These findings are similar to the present study confirming that the stability of 3D printing increased when there was a cross-arch stabilization structure, and accordingly it shows the necessity for a cross-arch stabilization structure in the 3D printing process of the model. 

Park et al. [38] performed a linear analysis of U-shaped models printed using two types of resin with the same DLP printer, and for both models the minimum deviation occurred in the front of the model and with contraction in the molar region. The median trueness values did not differ significantly between the two DLP groups, but the distribution of the values in the lower 50% relative to the median value has varied markedly with the resin type, which means that the trueness differed with the resin type. This reflects the contraction of the posterior teeth according to the resin type, and can be interpreted as the stability of 3D printing increasing when a cross-arch stabilization structure is present, accounting the results obtained in the present study.

In this study, the accuracy was low for the printed models, with a 1.5 mm or 4 mm-thick shell and a hollow interior. These models require less photopolymer resin than the fully filled model in the 3D printing process, with the 1.5 mm hollow model consuming only 54.9–62.7% of resin required to produce the fully filled model; however, that model had the lowest accuracy. The presence or absence of the cross-arch plate also affected the accuracy, and it was thought that hollowing the model reduced the overall volume stability during the printing and post-curing processes [39]. Rungrojwittayakul et al. [31] compared the trueness and precision between the U-shaped models with hollow and solid internal structures and a shell thickness of 2 mm. An error of 45 μm was found for the model with a shell thickness of 2 mm produced by continuous liquid interface production (CLIP), while the error was 35 μm for the solid model. For models with a shell thickness of 2 mm printed using a DLP 3D printer the error was 77 μm, with the same error found for the solid model. This meant that there was no significant difference in the printing accuracy between the solid and hollow models produced using either a CLIP or DLP printer. That study showed a similar trend to our study, but since cross-arch bars were applied to all of the models in that study, there was a similarity between the results with the hollow and fully filled groups of Group P in which a cross-arch plate was applied. On the other hand, the absence of a cross-arch plate in the design with a U shape resulted in the same difference between our studies.

In this study, 3D printing was performed using a selective pixel application method in the roughly filled model. This technique utilizes a microscopic mirror during DLP printing, which is controlled at the software level so that the UV light does not cause excessive curing. When printing a model using a DLP 3D printer, large curing areas can be associated with UV-light leak on the edges of the cured 3D printed layer and the masking surface of the LCD screen, possibly resulting in overcuring, which increases the volume of the product [40,41,42]. In the present study, the interiors of the fully filled and roughly filled models were designed to be solid, and the range of the applied light source was wider than for the 1.5 and 4 mm hollow models. Therefore, if the selective pixel application method was not utilized, model deformation may have occurred during printing, and so this method was included in the study design. However, this study found very little difference in accuracy between the roughly filled and fully filled models, and the amounts of photopolymer resin consumed were almost the same. 

Moreover, the application of the optimal curing time per layer in 3D printing during the preparation process can explain why there was no difference in the overcuring accuracy between the selective pixel application model and the fully filled model in this study. The application of an appropriate design using the slicing software and the output equipment stage (e.g., for the curing times of the various layers) will optimize the accuracy of the model output. The trueness of the hexagon-filled model was not better than those for the fully filled and roughly filled models. However, the hexagon-filled model in Group U showed the best precision. Since the internal structure was reinforced with a hexagon structure and the density of the internal structure was lower, the most stable output environment was achieved during printing due to the appropriate UV exposure being applied. Yang and Huang [43], similarly, found very high stability for a model having an internal honeycomb structure, and another previous study found that an object containing an internal structure with appropriate porosity can produce accurate printing results and superior mechanical properties such as stability and strength [44]. The hexagon-filled model has the advantage of reducing resin consumption, but the design of this internal structure requires a complicated modeling process and did not show higher accuracy in the trueness evaluation, and so it seems that there are not many advantages in using this model.

This study found that the cross-arch stabilization plate structure plays a very important role in producing a high-accuracy dental model, and modeling in a solid form is superior to hollow modeling despite requiring the use of more resin. 3D printed models can be used in the dental clinical environment not only for consultation and diagnosis, but also for prosthesis production, and an appropriate production method needs to be determined depending on the intended purpose. The most cost-effective approach would be to reduce resin consumption by making a U-shaped 1.5 mm hollow model for consultations. However, for fabricating an accurate prosthesis, the use of a cross-arch plate and the fully filled model design that involves completely filling the interior appears to be the best approach.

Further studies are needed for applications that were not covered in this study. Since this study focused on the modeling conditions, analyses were conducted with one type of 3D printer, and various types of photopolymer resin were not used. Future investigations of the 3D printing process using various printing methods and types of resin with various components would provide wider insights. It is also worth studying the effects of using various types of equipment with various light sources, wavelengths, and temperatures in the post-curing stage after the 3D printing process, including changes in the volume of the final products.

## 5. Conclusions

The trueness error was larger in the U-shaped group than in the cross-arch plate group in this study, and was the largest in the 1.5 mm group, while it was smallest for the roughly filled and fully filled models in the cross-arch plate group. This precision was not stable in the U-shaped group, whereas it was stable in the cross-arch plate group. Meanwhile, the output stability was highest for the hexagon-filled model in the U-shaped group, and the lowest for the 4 mm model in the cross-arch plate group.

## Figures and Tables

**Table 1:** Resin consumption according to model design (units: mL).

Each Name	Cross-Arch Plate (Group P)	U-Shaped Arch Plate (Group U)
Hollow model with 1.5 mm-thick shell	12.28	8.83
Hollow model with 4 mm-thick shell	17.38	13.89
Hexagon-filled model	15.22	13.48
Roughly filled model	19.50	15.59
Fully filled model	19.56	16.07
